# The role of ion channels in T cell function and disease

**DOI:** 10.3389/fimmu.2023.1238171

**Published:** 2023-08-29

**Authors:** Nicholas Manolios, John Papaemmanouil, David J. Adams

**Affiliations:** ^1^ Faculty of Medicine and Health, The University of Sydney, Sydney, NSW, Australia; ^2^ Department of Rheumatology, Westmead Hospital, Sydney, NSW, Australia; ^3^ Illawarra Health and Medical Research Institute (IHMRI), Faculty of Science, Medicine and Health, University of Wollongong, Wollongong, NSW, Australia

**Keywords:** ion channels, calcium signalling, T cells, antigen receptor, signal transduction, therapeutics

## Abstract

T lymphocytes (T cells) are an important sub-group of cells in our immune system responsible for cell-mediated adaptive responses and maintaining immune homeostasis. Abnormalities in T cell function, lead the way to the persistence of infection, impaired immunosurveillance, lack of suppression of cancer growth, and autoimmune diseases. Ion channels play a critical role in the regulation of T cell signaling and cellular function and are often overlooked and understudied. Little is known about the ion “channelome” and the interaction of ion channels in immune cells. This review aims to summarize the published data on the impact of ion channels on T cell function and disease. The importance of ion channels in health and disease plus the fact they are easily accessible by virtue of being expressed on the surface of plasma membranes makes them excellent drug targets.

## Introduction

All cells of the human body have and need membrane ion channels to allow anions and cations to move across the lipid membranes, provide homeostasis, cellular activation, and function (1). Ions are crucial for maintaining the integrity, identity, and stability of cells and supporting electrical potential across membranes. This is particularly important for cells such as muscle and neurons, which have the added function of being able to regulate membrane voltage between the extracellular and intracellular compartments allowing the cells to transmit electrical signals necessary for cardiac rhythm and nerve impulses, respectively. Within the last few decades, there has been increased awareness and recognition of the critical role played by ion channels and transporters in immunity (2). Foremost, much of this attention has arisen from T cell studies identifying a functional network of at least five major ion channels that include calcium (Ca^2+^) release-activated Ca^2+^ (CRAC) channels, purinoreceptor (P2X) receptors, transient receptor potential (TRP) channels, potassium (K^+^), and chloride (Cl^-^) channels. These ion channels have been extensively studied (3) and the field is evolving as new studies continue to identify novel channels (4), assembly intermediates, binding sites, and functions (5) in T cells. Although there are significant differences in ion channel expression and usage between mice and humans this review does not critically distinguish between the two but tries to give an overview of their importance in both.

## Ion channels expressed on T cells

**Figure 1:**
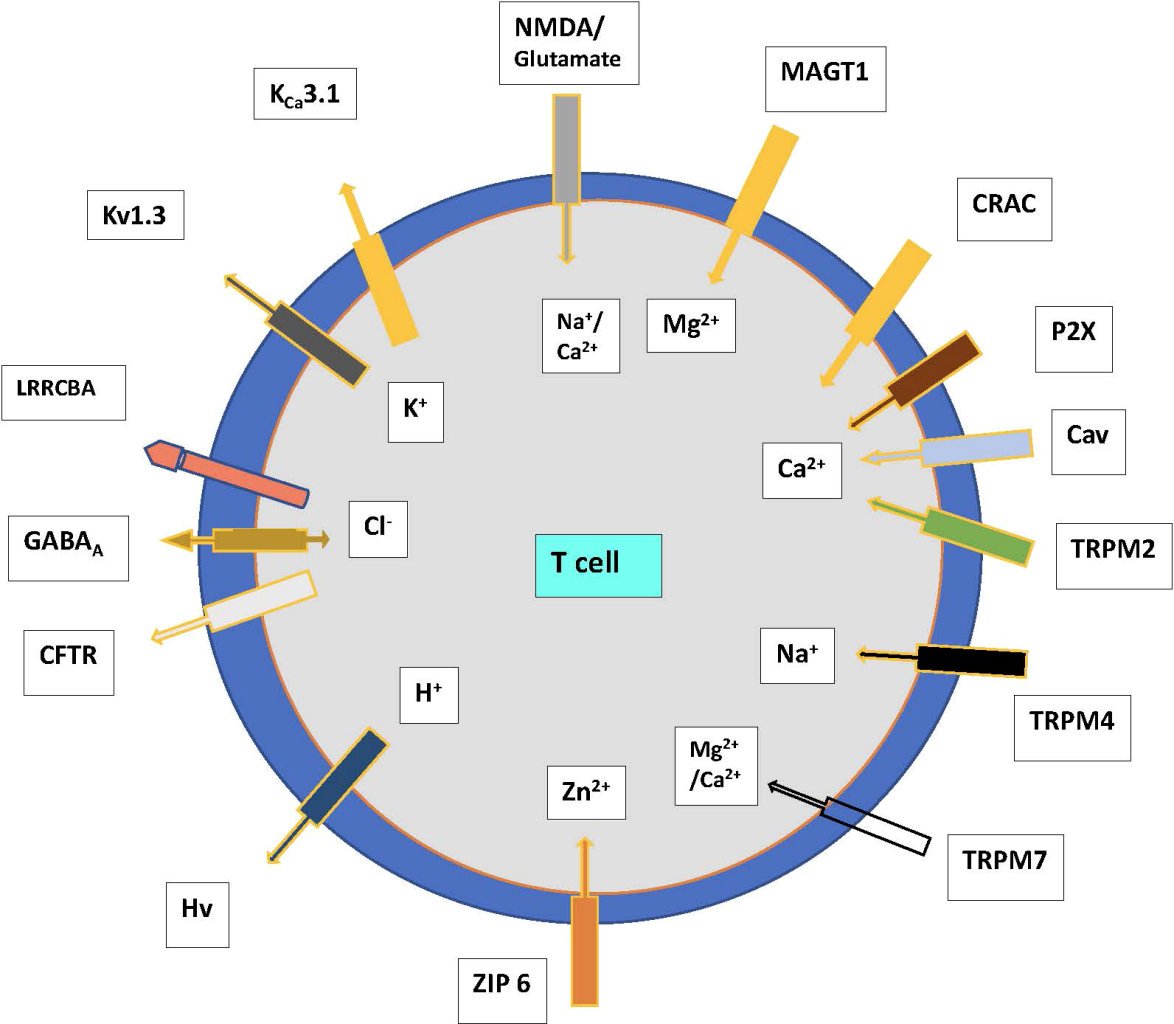
Ion channels and transporters showing the flow of cations and anions in T cells. *Abbreviations*. CFTR—cystic fibrosis transmembrane conductance regulator, CRAC—Ca^2+^ release-activated channel, GABA—γ-amino butyric acid, P2X—purinergic receptor, T—T cell, TRP—transient receptor potential family, leucine-rich repeat-containing 8 protein (LRRC8) receptors eg LRRCBA. N-methyl-D- aspartate (NMDA); Magnesium transporter protein 1 (MAGT1); Voltage-gated proton channels (Hvcn1); TRP mulcolipin (TRPM), ZIP 6, zinc transporter.

The major ion channels and transporters that modulate the influx of Ca^2+^, K^+^, sodium (Na^+^), magnesium (Mg^2+^), and zinc (Zn^2+^) which maintain electrical gradients and contribute to cell survival are outlined in **Figure 1**. Anions such as Cl^-^ are regulated by the cystic fibrosis transmembrane conductance regulator (CFTR) and the γ-aminobutyric acid type A (GABA_A_) receptors, which play a role in the movement and acidification of organelles (6). The volume and size of cells are regulated by the volume-regulated Cl^-^ or anion current (VRAC or Cl_swell_) channels that open upon swelling of T cells in a hypotonic environment, resulting in the efflux of Cl^-^, and ultimately water from the cell, returning the cell volume to normal. The function of this network of ion channels is not stagnant and can adapt depending on the state of cellular activation and differentiation to allow different types of immune responses to occur (7). The known and most studied ion channels on T cells are described below.

### Calcium channels

Ca^2+^ plays a critical role in many intracellular events. Ca^2+^ cannot diffuse across plasma membranes and has to enter cells *via* Ca^2+^ pores or be released from intracellular stores found at the endoplasmic reticulum (ER) and mitochondria. Ca^2+^ ions are released from the ER stores by a process known as store-operated Ca^2+^ entry (SOCE) (8). Depletion of luminal ER Ca^2+^ stores is sensed by the stromal interaction molecule (STIM) 1 localized in the ER, which then translocates, binds, and oligomerises with another protein Orai1, a Ca^2+^ selective ion channel protein on the cell surface membrane, conveying the signal from the ER to the plasma membrane to activate CRAC channels. CRAC channels are composed of three proteins, Orai1, Orai2, and Orai3 (3, 8–12). The Orai-STIM complex is a channel, present in most immune cells, with high Ca^2+^ selectivity that can generate a sustained Ca^2+^ signal to increase intracellular Ca^2+^, critical to the activation of calcineurin and the transcription factor, nuclear factor of activated T cells (NFAT), which activates the expression of cytokine genes encoding tumor necrosis factor (TNF), interleukin-2 (IL-2), and IL-6 (13). Calcium signalling and the relationship between SOCE and CRAC channels are comprehensively reviewed by Prakriya and Lewis (14), and Trebak and Kinet (15), respectively. To maintain Ca^2+^ balance in T cells, transport of calcium out of the cell is necessary and regulated by plasma membrane Ca^2+^ ATPase (PMCA).

Voltage-gated calcium (Ca_v_1) channels are expressed by T lymphocytes and function in a T cell receptor (TCR) stimulation-dependent and voltage-independent manner (16). There are two types of channels, the high voltage-activated (L-type) and the transient, low voltage-activated (T-type) Ca^2+^ channels which are thought to fine-tune TCR-mediated Ca^2+^ signals (15). Recently, Erdogmus et al. (5) have demonstrated that Cavβ1 regulates T cell expansion and apoptosis independently of voltage-gated Ca^2+^ channel function.

### Potassium channels

A critical feature that allows Ca^2+^ to influx into the cell is the ability to maintain electrochemical stability by effluxing a similarly charged molecule. Potassium channels modulate cellular homeostasis by controlling the efflux of K^+^ leading to hyperpolarization of the cell and inhibiting membrane depolarization induced by the influx of ions such as Ca^2+^ and Mg^2+^. With the increased influx of Ca^2+^, the cell membrane potential becomes more positive, a term called “depolarization”, which causes a significant electrochemical variance between the extracellular and intracellular compartments. As a result, K^+^ ions move out of the cell through the lymphocyte K^+^ channel identified as K_v_1.3. This channel is voltage-dependent and changes conformation when the cell is depolarized leading to an open channel discriminatory for K^+^ ions (17). A second type of K^+^ channel exists to efflux K^+^ termed K_Ca_3.1 (Ca^2+^-activated potassium channel), which is not dependent on voltage change, but a rise in cytosolic Ca^2+^. K_Ca_3.1 is closed under resting conditions but opens rapidly if the intracellular Ca^2+^ concentration rises. Both cation channels modulate cellular homeostasis by controlling the efflux of K^+^ leading to hyperpolarization of the plasma membrane and inhibiting membrane depolarization induced by the influx of ions such as Ca^2+^and Mg^2+^. K_v_1.3 and K_Ca_3.1 are similar in structure but differ in the mechanism of activation (18). K_v_1.3 is activated by membrane depolarization leading to K^+^ efflux, whereas K_Ca_3.1 activation requires the release of Ca^2+^ and calmodulin before activation.

### Purinoreceptor (P2X) channels

The P2X receptor-channel is a family of proteins that include seven subunits (P2X1-7), which are located on the cell surface and are ligand (ATP)-gated ion channels that allow the influx of Na^+^, Ca^2+^, and other cations (19). In humans, at least three different ionotropic P2X receptors (P2X1, P2X4, and P2X7) have been linked to Ca^2+^ influx (20, 21). P2X7 causes Ca^2+^ influx and activation of calcineurin, resulting in the activation, proliferation, and IL-2 production of T cells. It has been suggested that P2X receptors regulate T cell immune responses *via* autocrine ATP signaling to amplify weak TCR signals, gene expression and T cell effector function (22). In the presence of high levels of extracellular ATP, the activity of P2X7 receptors results in cellular apoptosis (23).

Another family of receptors called metabotropic receptors are largely monomeric proteins with an extracellular domain that contains a binding site and an intracellular domain that binds to existing G protein-coupled receptors (GPCRs) and second messengers, to indirectly modulate ion channel activity. Metabotropic purinoceptors (P2Y) comprise eight subtypes (P2Y1/2/4/6/11/12/13/14) and are expressed on T cells. T cell activation with the P2Y6 receptor ligand, uridine diphosphate (UDP), leads to TCR-dependent elevation of intracellular Ca^2+^ concentration whereas inhibition of P2Y6 receptors by the selective antagonist MRS 2578, inhibits CD25 expression, IL-2 production, and cytoplasmic Ca^2+^ levels in T cells (reviewed by Wang (24)). A variety of GPCRs and their signalling mediators (G protein coupled receptor kinases, regulators of G protein signalling, and β-arrestin), are expressed in T cell and involved in T cell-mediated immunity. Although not ion channels *per se*, GPCRs couple to ion channels and receptors such as purinergic P2Y6, to mobilize and raise the intracellular Ca^2+^ concentration (24).

### Transient receptor potential channels

This family of six subtypes with twenty eight members of channels are non-selective and permeable to Ca^2+^, nickel (Ni^2+^), Zn^2+^, Mg^2+^, and Na^+^ (25). In humans, six subfamilies include TRP canonical (TRPC), TRP melastatin (TRPM), TRP ankyrin (TRPA), TRP vanilloid (TRPV), TRP mulcolipin (TRPM), and TRP polycystic (TRPP) (25, 26). In addition to the CRAC channel these ion channels also mediate Ca^2+^ signals in T cells. TRP channels are activated by increases in intracellular Ca^2+^ that negatively modulate SOCE and promotes Na^+^ influx, membrane depolarization, and a reduction in the electrical driving force for Ca^2+^ influx, thus providing a negative feedback mechanism to prevent Ca^2+^ overload in cells (27–29). TRPM8 has been associated with T cell activation, increased CD25 and CD69 expression, TNF-α secretion, and T cell proliferation (29). TRPM4 channels are permeable to Na^+^, K^+^, and partially to Ca^2+^ (28).

### Anion channels

Volume-regulated Cl^-^ or anion current (VRAC or Cl_swell_) channels open when T cells distend in a hypotonic milieu, resulting in the efflux of Cl^-^, and water, returning the cell volume back to normal (3, 7, 30). K_v_1.3 and Cl_swell_ work together to regulate T cell volume. VRACs channels expressed by T cells include GABA receptor, CFTR, and leucine-rich repeat-containing 8 protein (LRRC8) receptors which play a critical role in cellular osmoregulation to prevent cell death. VRACs channels are composed of the obligatory subunit LRRC8A (31) and promote the efflux of Cl^-^ from the intracellular to the extracellular compartment. Other roles include inhibition of function and proliferation of T cells, increased IL-4 secretion of CD4 cells, decreased IL-2 and TNF production on CD8 cells, and T cell development, function, and survival (32). Recently, LRRC8C has been shown to be essential for VRAC function in T cells, regulating T-cell function through the LRRC8C-STING-p53 signaling pathway (4). This represents a new inhibitory pathway in T cells that controls T cell function and adaptive immunity (4).

### Transporters and other ion channels

Ion channels are membrane pores that passively allow ions to move quickly from higher concentrations to lower concentrations. Transporters, by contrast, are membrane proteins that change shape and allow ions to be carried from one compartment to another of the membrane. Consequently, transporters are energy dependent, slow, and can move ions/molecules against their concentration gradient (from lower to higher concentration). The divalent ion Mg^2+^ is an important molecule required for T cell function requiring a transporter to enter the cells (7). Magnesium transporter protein 1 (MAGT1) is highly specific for Mg^2+^ and does not transport Ca^2+^, Zn^2+^, or Ni^2+^ (33). This MAGT1 transporter has a role in T cell development and function (34). Interestingly, membrane receptors for neurotransmitters have also been recognized in T cells. These include α-amino-3-hydroxy-5-methyl-4-isoxazole propionic acid (AMPA) and N-methyl-D- aspartate (NMDA) which are ionotropic glutamate receptor-channels. They are non-selective cation channels that are largely permeable to Na^+^ and K^+^ and to a lesser extent Ca^2+^ (35). Other ligand-gated receptors expressed in T cells include muscarinic and nicotinic acetylcholine receptors (36).

Voltage-gated proton channels (Hvcn1) reduce cytosol acidification and facilitates the production of reactive oxygen species. These channels only allow protons and no other ions to cross cell membranes (37). Intracellular acidification plays an important role in modulating the function of lymphocytes. Low intracellular pH reduces the proliferation and function of human and mouse T cells. Restoration of pH to physiological levels rescues T cell function. Deletion of Hvcn1 receptors leads to increase intracellular acidification, decreased expansion, loss of effector function and apoptosis in CD4+ cells. In CD8+ cells there is decreased expansion, loss of effector function, mitochondrial damage, AMPK activation and metabolic adaptation (38). Hvcn1 plays an important role in controlling intracellular acidification in T cells during the differentiation of T cells during the transition from naive to activated T cells (38, 39).

### Impact of ion channels on T cell function

Intracellular Ca^2+^ concentrations control the regulation and function of transcription factors that ultimately influence the differentiation and effector tasks of T cells (11, 15, 39), **Table 1**. The temporal and spatial fluctuations of intracellular Ca^2+^ have important consequences on T cell physiology and can contribute to the generation of different signalling pathways, the development of heterogeneity in T cells, the maintenance of immunological tolerance, and prevention of autoimmunity (10, 52). Dolmetsch et al. (53) were first to show that modulation of the amplitude and frequency of Ca^2+^ fluxes caused a disparity in the activation of transcription factors, c-Jun NH_2-_ terminal kinase (JNK), NFAT, and NF-κB. This differential “decoding” or sensitivity of these transcription factors means that the amount and timing of Ca^2+^ can regulate and govern an appropriate response. NF-kB and JNK were selectively activated by a large transient intracellular rise of Ca^2+^, whereas NFAT was activated by a low, sustained Ca^2+^ plateau. The disparity in activation resulted from differences in the Ca^2+^ sensitivities and kinetic behavior of the three pathways. These findings provide a mechanism by which Ca^2+^ can achieve specificity in signalling to the nucleus. Although these results were generated from B cells there is no reason to suspect that a similar mechanism does not exist in T cells.

**Table 1:** Functional effects of store-operated Ca2^+^ entry (SOCE) on T cells.

Controls T cell proliferation (2, 7, 14, 15)	
T cell migration (40, 41)
Apoptosis (42)
Cytokine production (43–45)
Transcriptional regulation (43–45)
Controls the development and function of T cells (2, 7, 14) /T _regs_ (10, 46, 47)
Controls proliferation, and expansion of CD4^+^/CD8^+^ cells (9)
Regulates function of TH17 cells (10, 47, 48)
Controls germinal center reaction and humoral immunity (49, 50)
T_regs_ development in the thymus (43, 51)

Ion channels by regulating ion movement and homeostasis influence T cell function. However recent evidence suggests that ion channels can regulate T cell function independent of their activity. Both K_v_1.3 and K_Ca_3.1 are recruited to the immunological synapse where it is possible that in this proximity with many other receptors they interact and couple with other molecules co-localised to the region (3, 37). Erdogmus et al. (5) have identified that the channel Cavβ1, encoded by *Cacnb1*, is a regulator of T cell function. Using patch clamp electrophysiology and intracellular Ca^2+^ measurements, they were also able to demonstrate that although Cavβ1 regulates T cell function, the enhanced apoptosis and impaired T cell expansion were independent of voltage-gated Ca^2+^ channel activity.

**Figure 2:**
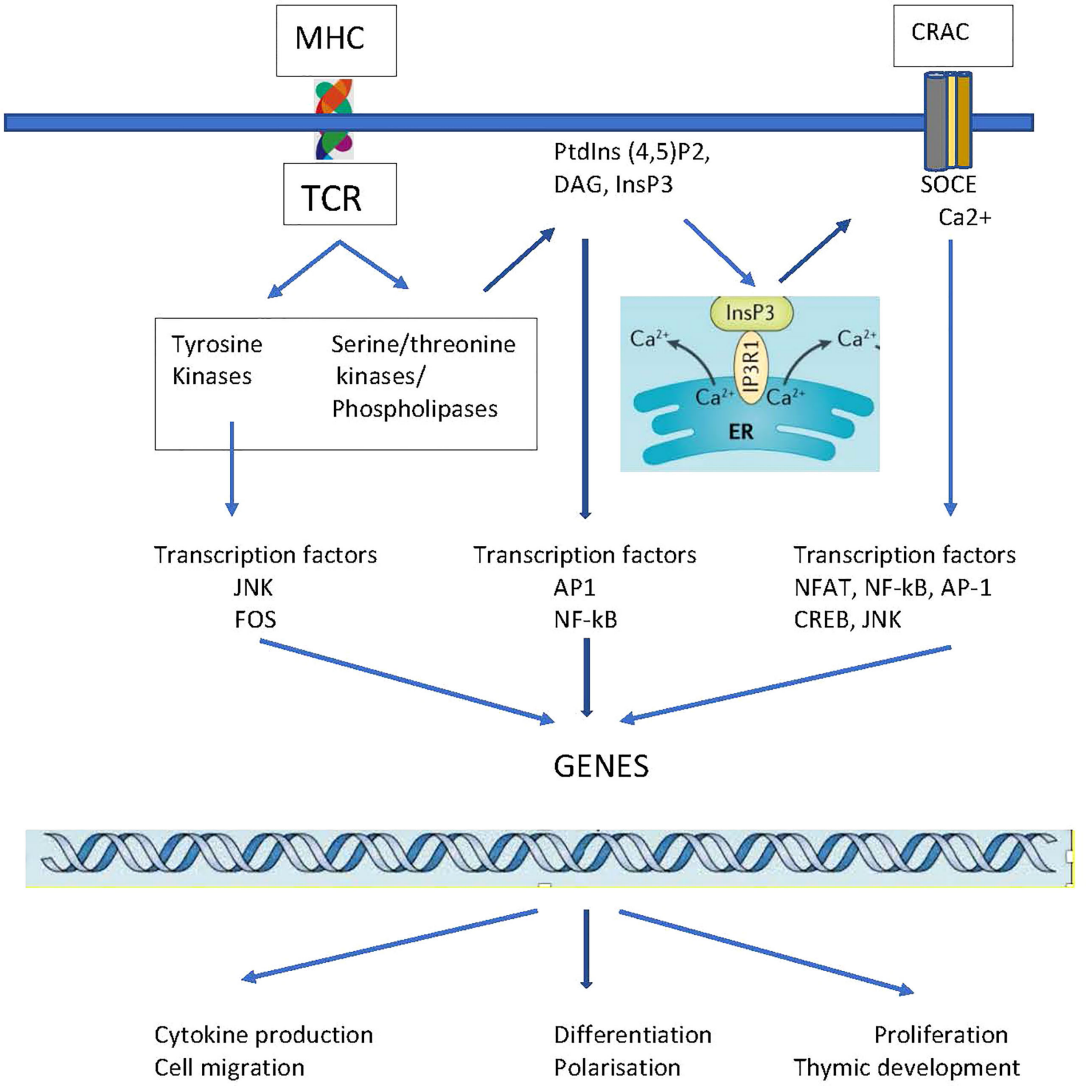
Overview of transcriptional events following TCR signal transduction. T cell activation results in signal propagation *via* three major signalling pathways: the Ca^2+^–calcineurin, mitogen-activated protein kinase (MAPK), and NF-κB signalling pathways. The concerted activity of these pathways, lead to the activation of transcription factors (Jun, Fos, AP-1, NF-κB, NFAT) that results in T cell proliferation, migration, cytokine production and effector functions. *Abbreviation*: phosphatidylinositol 3-kinase (PI3K), CRAC, Ca^2+^ release-activated Ca^2+^ channel; DAG, diacylglycerol; ER, endoplasmic reticulum; InsP3, inositol trisphosphate; JNK, Jun N-terminal kinase; PLCγ1, phospholipase Cγ1; PtdIns(4,5)P2, phosphatidylinositol 4,5-bisphosphate.

Upon TCR recognition of antigen, a cascade of coordinated phosphorylation events occur that involve many signalling proteins and ion channels (54). Protein kinases result in the activation of phospholipase C (39, 55) hydrolyzing phosphatidylinositol bisphosphates (PIP_2_), into soluble inositol 1,4,5- triphosphate (IP_3_) and diacylglycerol (DAG) that is membrane-bound (56). DAG activates Ras/Raf-1/MEK/ERK to stimulate the transcription factor nuclear factor-κB (NF-κB) that results in cytokine and chemokine gene transcription (54). IP_3_ binds to the IP_3_ receptor, expressed in the ER, which releases Ca^2+^ from the ER stores (8) into the cytoplasm by the process of SOCE. Within minutes, this results in a large influx of Ca^2+^ required for refilling Ca^2+^ (2) stores *via* PMCA pumps (13) and to support downstream Ca^2+^ required events for signalling. After TCR stimulation CRAC channels influx Ca^2+^ which becomes available to regulate the function of serine/threonine-phosphatase calcineurin, NFAT, and NF-κB. Ca^2+^-bound calmodulin then results in the activation of transcription factor cAMP responsive element binding protein 1 (CREB1) that regulates IL-2 and other diverse cellular events, including proliferation, survival, and cellular differentiation (39, 57), **Figure 2**. The molecular mechanism by which SOCE and CRAC channels regulate T cell proliferation (43), apoptosis (42), cytokine production (44, 45) and migration (40, 41) is outlined in **Table 1**. The secondary cellular events post TCR activation include:

i. Proliferation. As previously discussed, K_v_1.3, and K_Ca_3.1 efflux K^+^ from T cells (7, 58). Pharmacological inhibition of these channels and CRAC prevent T cell proliferation. Blocking only K_v_1.3 alters the secretion of cytokines by CD4 T cells (59). The decrease in IL-2 production may correlate with the inhibition of T cell proliferation.

ii. Polarization. The nature of the antigen influences whether Th1, Th2, or Th17 responses are utilized for its elimination. The T cell profile developed is dependent on the type of cytokine released which is influenced by ion channels. For example, the expression of T*-bet* and IFN-γ (60) is increased in the absence of TRPA1 causing the polarization of T cells into a Th1 profile. T cell profiles toward a Th2 response have been associated with the upregulation of Cav channels (7). The entry of Ca^2+^ through the P2X7 receptor activates ERK1 or ERK2, which suppresses the transcription of *forkhead box P3* and promotes the polarization of T cells toward a Th17 phenotype, altogether inhibiting the regulatory T cells (Treg) (7).

iii. Ion channel phenotype changes in lymphocyte subsets. As shown, the modulation of intracellular Ca^2+^ levels regulate the Th1 and Th2 differentiation of T cells (27) and establishes central and effector memory phenotypes in T cells (18). T cells express different ion channel patterns based on their level of T cell activation and the degree of differentiation. This difference gives rise to different T cell subtypes based on their ion channel expression. CCR7+CD45RA+ naive human T cells predominantly express K_v_1.3 channels. Upon antigen activation, they upregulate K_Ca_3.1 expression (7). In Th1, Th2 and central memory T cells (T_CM_) K_Ca_3.1 is selectively upregulated upon activation (7). In contrast, Th17 and effector memory T (T_EM_) cells selectively upregulate K_v_1.3 channels on activation. This contrast makes Th17 and T_EM_ cells uniquely susceptible to K_v_1.3 channel blockade, whereas other T cell subsets are consequently spared. In addition to K^+^ channel expression during early T cell activation there is an upregulation of STIM1 and Orai1 within days. This ensures CRAC channel-mediated Ca^2+^ signalling for cytokine gene expression and differentiation.

iv. Cytokine secretion. Ca^2+^ influx through the CRAC channels regulates three cytokine pathways: (a) NFAT signalling; (b) NF-κB pathway, and (c) c-Jun terminal kinase (JNK) pathway (43), **Figure 2**. The NFAT pathway, also known as the calmodulin–calcineurin pathway, requires the low and extended Ca^2+^ influx to activate calcineurin which then dephosphorylates NFAT promoting its translocation to the nucleus, and production of IL-2 (43). Unlike NFAT a high and short burst of Ca^2+^ in the cytoplasm is required to activate calcineurin that then regulates the NF-κB signalling pathway (56). Another pathway related to Ca^2+^ influx is the JNK pathway. Activation of c-Jun along with calcineurin, triggers the activator protein 1 (AP-1) transcription factor complex, which plays a role in cell growth and IL-2 induction (43). The combined coordinated activation of NFAT, NF-kB, and AP-1 induces the production of cytokines, including IL- 1β, IL-2, IL-4, IL-6, IL-8, IFN-γ, and TNF-α.

v. Migration. Using a dominant-negative Orai1-E106A mutant to suppress Ca^2+^ signalling Greenberg et al. (40) demonstrated that CRAC channel-mediated Ca^2+^ influx was necessary for T cell homing to lymph nodes. In T cells, uropods which facilitate cell motility and chemotaxis, express K_Ca_3.1 and TRPM 7 which can regulate the migration of human T cells (61).

In the thymus, SOCE is essential for Treg development. Mice deficient in SOCE have a reduced number of peripheral and thymic Treg cells (51, 46). In addition to the CRAC channels, TRP channels are involved in the regulation of intracellular Ca^2+^ homeostatis (28). These channels include TRPM2, TRPV1 and TRPM7 the function of which includes thymic development and the production of thymocyte growth factors (60, 62, 63). In mice, TRPM7 deletion impairs T cell development and function (64). Extracellular ATP binding to P2X4 and P2X7 mediates Ca^2+^ influx that can regulate T cell migration, inhibit regulatory T cell differentiation, promote Th1, Th17 (65) cell development, and stimulate the establishment and maintenance of (tissue-resident) memory CD8+ T cells in mice (66, 67). The role of CRAC channels in immunity and the molecular mechanisms by which CRAC channels can control the function of various T cell subsets, including T follicular helper (Tfh) cells are reviewed by Vaeth et al. (10).

## The role of ion channels in disease

Channelopathies is the term given to an assorted group of disorders resulting from ion channel dysfunction. These abnormalities may be congenital due to genetic mutations or acquired factors (6, 68). The term CRAC channelopathy refers to a complex clinical phenotype caused by loss-of-function mutations in Orai1 and STIM1 in humans. The condition is characterized by immunodeficiency, autoimmunity, and non-immunological symptoms such as pain and fatigue. In the human genome there are more than 400 genes that encode ion channels. Mutations in these genes can manifest as very rare disorders which are life-threatening to mild and disabling conditions, **Table 2**. The organs predominantly affected include the nervous, cardiovascular, respiratory, endocrine, musculoskeletal, and immune systems causing autoimmune diseases or immunodeficiencies. As molecular-genetic and electrophysiological studies advance new disorders are being recognised (86, 81, 82).

**Table 2:** Properties and functions of ion channels and transporters in T cells.

Channel			Ion		Function	Channelopathy	
Ca^2+^							
CRAC							
Orai 1			Ca^2+^		Involved with T cell activation, proliferation, cytokine production 7, 14, 15	Mutations in STIM1, ORAI, 69 Leads to combined immunodeficiency 68, autoimmunity 70, 71
Orai 2						
Orai 3						
TRP							
TRP			Ca^2+^, Na^+^		Enhances TCR signalling Regulates Th1/Th2 differentiation, 26	
TRPM2			Ca^2+^, Na^+^		Activation and cytokine production	
TRPM4			Na^+^		Cytokine production 29	
TRPM7			Ni^2+^,Zn^2+^, Mg^2+^>Ca^2+^		Thymocyte development, production of thymic growth factors 7		
P2X							
P2X7			Ca^2+^, Na^+^ other		Involved with T-cell proliferation, cytokine production,	SLE 23, 72, 73
					Promotes Th17, Inhibits Treg differentiation 19		
P2X1,4			Ca^2+^, Na^+^		T-cell proliferation, Cytokine production, Thymocyte apoptosis		
Cav							
Cav, 1.2, 1.3, 1.4			Ca^2+^,		Cytokine production, CD8 T-cell survival, CD8 T-cell immunity to infection, Th2 function in asthma	
					Secretion of IL-2.	
K^+^							
K_v_1.3			K^+^		Regulation of V_m_ , T-cell activation (Th_17_, T_EM_) Cytokine production,	Multiple sclerosis 74, 75 Congestive cardiac failure 76 Autoimmunity and inflammation 70	
K_Ca_3.1			K^+^		Hyperpolarisation of Vm, T-cell activation (Th_1_, Th_2_, TCM), cytokine production, autoimmune colitis	Cancer 77–79
Chloride							
Cl_swell_			Cl^-^ (I^-^, Br^-^)		Apoptosis in T-cells	Agammaglobulinemia 80
CFTR			Cl-		Cytokine production	
GABA_A_			Cl-		Inhibition of T-cell proliferation, cytokine production, cytotoxicity, and T-cell mediated autoimmunity	
Magnesium							
MagT1			Mg^2+^		CD4+ development and activation. Immunity to infection (EBV) 49	XMEN syndrome caused by X-linked mutations in *MAGT1* 33
Zinc							
ZIP3, ZIP6, ZIP8					T-cell activation, T-cell development 7	Acrodermatitis enteropathica with immune deficiency caused by mutations in intestinal *ZIP4* transporter
Other							
Nicotinic Acetylcholine Receptor			Allows entry of cations		Stabilises open and desensitised state of channel	
Glutamate receptor eg NMDA			Na^+^, Ca^2+^			
Acid sensing ion channel			H+ Transports H_2_O and glycerol in some cases		Regulates fluid balance, osmotic potential	

Cav, voltage-gated Ca^2+^ channel; CFTR, Cystic fibrosis transmembrane conductance regulator; CRAC, Ca^2+^ release activated Ca^2+^ channel; GABA, γ-aminobutyric acid; Kv, voltage-gated K^+^ channel; KCa, Ca^2+^ gated K^+^ channel; MagT, Mg^2+^ transporter; STIM, stromal interaction molecule; TRP, transient receptor potential; Vm, membrane potential; XMEN, X-linked immunodeficiency with Mg^2+^ defect and EBV infection and neoplasia; ZIP, Zrt-Irt like protein; ZnT, Zinc transporter.

### Immunodeficiencies

Patients with mutations in the Orai1 or STIM1 symptoms have reduced numbers of Treg cells and suffer from a complex CRAC channelopathy syndrome that causes severe immunodeficiency. Given the Orai-STIM complex’s role in Ca^2+^ influx, dysfunction in T cell activation, proliferation, and production of cytokines are believed to be the underlying mechanism behind this immunodeficiency (69). Similarly, patients with an inherited mutation of the gene encoding the magnesium channel, MAGT1, suffer a rare form of immunodeficiency known as X-linked immunodeficiency with magnesium defect (XMEN), EBV infection, and neoplasia (33). Little is known as to the role and function of MAGT1 in T cell development and activation, and how a mutation in this channel causes disease. In addition to its role in VRACs, the LRRC8 protein family is also associated with agammaglobulinemia (80).

### Autoimmune diseases

SOCE can regulate immune tolerance and autoimmunity by controlling the function of Treg and Th17 cells, reviewed in detail by Vaeth et al. (10). Other ion channels such as the human P2X7 receptor has been shown to mediate ATP-induced apoptotic death of T cells. Treg’s overexpress P2X7 receptors during differentiation. Activation of the P2X7 receptor inhibits Treg function and, when IL-6 is present, promotes conversion into Th17 cells (65). ATP binds to P2 receptors and its levels are regulated by NTPDase (CD39). CD39 is upregulated in systemic lupus erythematosus (SLE) patients who exhibit increased levels of ATP that binds to P2X receptors resulting in activation of the inflammasome, release of IL-1β, and other cytokines associated with disease pathogenesis (72). Considering P2X7 stimulation is proinflammatory and induces apoptosis, the question arose of whether. functional polymorphisms in this gene could affect lupus susceptibility. Forchap et al. (73) investigated the role of the P2X7 receptor and its loss-of-function Glu496Ala (rs3751143) polymorphism (SNP) in the development of SLE. A loss-of-function SNP at position rs3751143 of the P2X7 gene did not appear to be a susceptibility gene locus for the development of sporadic SLE. However, this study does not exclude a connection between P2X7 channels and SLE pathogenesis (41). Interestingly, in patients with SLE, the Tfh cell response to extracellular ATP *ex vivo* was reduced when compared to healthy individuals. It is suggested that the reduced expression of P2X7 channels observed in SLE patients may serve to limit the apoptosis and encourage Tfh T cell subset activation (65, 72). The findings by Faliti et al. (23), examining the role of P2X7 receptors in Tfh cells of patients, showed human Tfh-cells from patients with SLE, when compared with healthy controls, displayed reduced sensitivity of P2X7 to ATP-mediated stimulation and consequently, reduced P2X7-dependant cell death. The Tfh cells of patients with SLE were found in larger numbers, had reduced P2X7R mRNA, greater germinal center reactions, IgA secretion and binding to commensals (23). These results were not observed in patients with primary anti-phospholipid syndrome or rheumatoid arthritis supporting the view that P2X7 receptor activity plays a role in SLE pathogenesis. Given that this review is an introduction to the key ion channels in T cells, other receptors such as P2X1 and P2X4 are not discussed.

### Infections

An important role of SOCE is to regulate the function of Th1, Th2 and Th17 cells that can provide adaptive immunity to viral, parasitic as well as bacterial and fungal infections (42). For recent reviews on the role of ion channels in T cell mediated immunity and viral infections see Vaeth et al. (10) and Bohmwald et al. (83).

### Cancer

Reduced KCa3.1 activity enhances the inhibitory effect of adenosine on the chemotaxis of T cells in cancer (77). Adenosine modulates immune responses by delaying the encounter of antigen-loaded dendritic cells with T cells by stimulating cAMP production and PKA1 activation through adenosine receptors (ARs) which inhibits KCa3.1 channel function, T cell proliferation, and cytokine release (79). Adenosine receptors comprise the P1 class of purinergic receptors and belong to the superfamily of GPCRs. ARs are classified into four subtypes, A_1_, A_2A_, A_2B_, and A_3_, which are activated by extracellular adenosine, and play a central role in the modulation of the immune system (84). Activation of the A_2A_ receptor enhances cyclic AMP production, which in turn activates PKA1, and phosphorylation of the transcription factor CREB.

In certain cancer patient populations, both K_Ca_3.1 activity and calmodulin expression are reduced, with the downregulation of membrane-proximal calmodulin being responsible for the suppression of K_Ca_3.1 activity and consequently limiting the ability of these T cells to infiltrate the adenosine-rich tumor microenvironment. It is believed the assembly and surface expression of K_Ca_3.1 channels are facilitated by calmodulin. In migrating human T cells, K_Ca_3.1 channels localize at the moving edge of the cell (uropod) where they control motility and chemotaxis. K_v_1.3 channels are associated with CD8 cytotoxic function. The tumour microenvironment, which is hypoxic and hyperkalemic, impairs the function of K_v_1.3 and K_Ca_3.1 which results in suppressed T cell motility and function. In mice, restoration of function and overexpression of K_v_1.3 channels restored T cell functionality, reduced tumor burden, and increased survival. Gawali et al. (78) demonstrated that treatment with both anti-PD1 (pembrolizumab) and anti-PD-L1 (atezolizumab) antibodies facilitated the function of K_Ca_3.1 and K_v_1.3 in head and neck squamous cell carcinoma (HNSCC) patients’ peripheral lymphocytes. This study suggests that immune checkpoint blockade improves T cell function by increasing K_Ca_3.1 and K_v_1.3 channel activity in HNSCC patients. In contrast, Markakis et al. (74) showed there was a more pronounced increase in functional K_v_1.3 expression in T cells from multiple sclerosis (MS) patients with secondary progressive MS status. K_v_1.3 channel up-regulation enhanced signalling in MS T cells enhancing Ca^2+^ availability through CRAC channels potentiating the immune stimulus, and heightening cytokine production, cytokine secretion, and proliferation. Moreover, the immune stimulus-induced K_v_1.3 activity in these cells, increases β1-integrin activation, facilitating their adhesion and cytokine-induced migration into the central nervous system.

The p53 tumor suppressor gene, plays a key role in controlling cell division and cell death. Activation of p53 prolongs cell-cycle arrest in G1, thereby preventing proliferation, and/or leading to apoptosis, mutations, or functional changes to p53 may result in cancer. Gosh et al. (85) have shown that p53 promotes the degradation of the DNA exonuclease TREX1, leading to the accumulation of cytosolic dsDNA which activates the cytosolic DNA sensor, cGAS, and its downstream effectors’ Stimulator of interferon genes (STING),/TBK1/IRF3 resulting in induction of type I interferons. The cGAS/STING pathway induces type I interferon production and is a key mediator of the innate immune system that functions to detect the presence of cytosolic DNA and, in response, induce expression of pro-inflammatory genes that lead to apoptosis or to the activation of defense mechanisms (86). Wild-type p53 activates cGAS/STING. Silencing of cGAS in cancer cells disables the innate immune response to the accumulation of cytosolic DNA. Conception et al. report that LRRC8C *via* cGMP uptake and suppression of Ca^2+^ influx, results in STING and p53 activation that acts as a negative regulator of T cell function that can impair adaptive immunity.

### Ion channels as pharmacotherapeutic targets

Ion channels are the primary target of approximately 10% of the marketed drugs and represent promising targets for the development of novel therapeutic agents (87). The Guide to Pharmacology 2021/22 by Alexander et al. (88) provides a concise overview of the key human drug targets. Links to the knowledgebase source of drug targets and their ligands are provided for more detailed views of target and ligand properties. A result summary of a patent search (https://patents.google.com/patent/US8357809B2/en; https://www.lens.org/lens/search/patent/list?q=ion%20channel%20inhibitors) using “ion channel inhibitors” as key words, over the last 5 years is shown in **Table 3**. These findings highlight the interest and application of ion channel inhibitors in clinical applications (89).

**Table 3:** Summary of patents filed over the last five years for drugs targeting ion channels.

PATENT ID	TITLE	LINK
US- 11008306- B2	Quinazolines as potassium ion channel inhibitors	https://patents.google.com/patent/US11008306B2/en
US-2022041552-A1	Charged ion channel blockers and methods for use	https://patents.google.com/patent/US20220041552A1/en
US-2019038573-A1	Ion channel activators and methods of use	https://patents.google.com/patent/US20190038573A1/en
US-2020101069-A1	CRAC channel inhibitors for the treatment of stroke and traumatic brain injury	https://patents.google.com/patent/US20200101069A1/en
US-2022193096-A1	Inhibitors of ncca-atp channels for therapy	https://patents.google.com/patent/US20220193096A1/en
JP-2021098713-A	Permanently charged sodium and calcium channel blockers as anti-inflammatory agents	https://patents.google.com/patent/JP2021098713A/en
US-2020108056-A1	Liposomal Mitigation of Drug-Induced Inhibition of the Cardiac IKR Channel	https://patents.google.com/patent/US20200108056A1/en
US-10774050-B2	Pyrimidines as sodium channel blockers	https://patents.google.com/patent/US10774050B2/en
US-10350430-B2	System comprising a nucleotide sequence encoding a volvox carteri light-activated ion channel protein (VCHR1)	https://patents.google.com/patent/US10350430B2/en
JP-2019142914-A	Trpa1 inhibitors for treating pain	https://patents.google.com/patent/JP2019142914A/en
US-11377438-B2	2-amino-n-heteroaryl-nicotinamides as Nav1.8 inhibitors	https://patents.google.com/patent/US11377438B2/en
US-10898496-B2	Targeting NCCa-ATP channel for organ protection following ischemic episode	https://patents.google.com/patent/US10898496B2/en
US-2019111137-A1	Methods for antagonists of a non-selective cation channel in neural cells	https://patents.google.com/patent/US20190111137A1/en
US-2022227732-A1	Pyridine carboxamide compounds for inhibiting Nav1.8	https://patents.google.com/patent/US20220227732A1/en
JP-6630004-B2	Macrocycles as Factor XIA inhibitors with aromatic P2 'groups	https://patents.google.com/patent/JP6630004B2/en
US-10668051-B2	Modulators of calcium release-activated calcium channel	https://patents.google.com/patent/US10668051B2/en
US-11357841-B2	Expansion of tumor infiltrating lymphocytes with potassium channel agonists and therapeutic uses thereof	https://patents.google.com/patent/US11357841B2/en
US-10701938-B2	Composition for inhibition of insect host sensing	https://patents.google.com/patent/US10701938B2/en
US-10653673-B2	Substituted imidazoles as N-type calcium channel blockers	https://patents.google.com/patent/US10653673B2/en
EP-3625214-B1	Deuterated pyridone amides and prodrugs thereof as modulators of sodium channels	https://patents.google.com/patent/EP3625214B1/en
US-11192863-B2	Antiviral compounds and methods	https://patents.google.com/patent/US11192863B2/en
US-10472359-B2	Pharmaceutical compositions comprising 4-((6bR,10aS)-3-methyl- 2,3,6b,9,10,10a-hexahydro-1H,7H-pyrido[3',4':4,5]pyrrolo[1,2,3-d e]quinoxalin-8-yl)-1-(4-fluorophenyl) butan-1-one for inhibiting serotoninreuptake transporter activity	
US-10472359-B2	https://patents.google.com/patent/US10472359B2/en	
JP-7072154-B2	Fertilizer pesticide product	https://patents.google.com/patent/JP7072154B2/en
US-10548886-B2	Methods of treatment using a JAK inhibitor compound	https://patents.google.com/patent/US10548886B2/en
US-2018172671-A1	Methods for treating neural cell swelling	https://patents.google.com/patent/US20180172671A1/en
ES-2882186-T3	JAK inhibitors containing a 4-membered heterocyclic amide	https://patents.google.com/patent/ES2882186T3/en
US-2020163902-A1	Therapeutic agents targeting the NCCA-ATP channel and methods of use thereof	https://patents.google.com/patent/US20200163902A1/en
AU-2018200632-B2	Cystathionine-y-lyase (CSE) inhibitors	https://patents.google.com/patent/AU2018200632B2/en
US-11439639-B2	Pyrazine-containing compound	https://patents.google.com/patent/US11439639B2/en

### Autoimmune diseases

T cell subsets express different ion channel phenotypes that provide the means to eliminate disease-specific autoantigen clones. The relative contribution of K_v_1.3 and K_Ca_3.1 related to their state of lymphocyte activation provides an opportunity to selectively target lymphocyte subsets for therapeutic purposes. Selective targeting of TEM cells in multiple sclerosis or diabetes mellitus type 1, where only pathogenic T cell clones have been noted to express high K_v_1.3, provides the opportunity to delete/inhibit disease-causing clones without compromising the overall immune system. For example, synovial fluid T cells from rheumatoid patients showed enhanced CCR7- and high K_v_1.3 expression, whereas T cells from osteoarthritis patients were CCR7+ with low K_v_1.3 expression. As such, inhibiting K_v_1.3 may provide a therapeutic target to modulate autoreactive TEM cells.

In animal models, inhibition of K_v_1.3 channels leads to less inflammation in experimental autoimmune encephalitis (EAE) and psoriasis. As opposed to other Kv channels, K_v_1.3 has only a minor impact on cardiac physiology. Therefore, considering the role of K_v_1.3 channels in TEM cells and their minimal impact on other organ systems, the channel is considered a prime target for pharmacotherapy. K_v_1.3 channel blockers have been found effective in the prevention and treatment of other conditions such as EAE, asthma, bone reabsorption in experimental periodontitis, pristane-induced arthritis, rheumatoid arthritis, multiple sclerosis, Type-1 diabetes mellitus, contact dermatitis, renal fibrosis, and delayed- type hypersensitivity reactions. This therapeutic effect is achieved through the specific suppression of TEM cells without compromising naïve and TCM cells that are unaffected (88). An important consideration of K_v_1.3 channel blocker monotherapy is that in many diseases, T cells are not the only cells involved. Consequently, it has been suggested that combination therapies be considered. For example, in rheumatoid arthritis a K_v_1.3 channel blocker may be paired with an ion channel blocker relevant to fibroblast-like synoviocytes to reduce both lymphocyte and fibroblast-like synoviocyte activity in affected joints.

A specific inhibitor of K_v_1.3 is the synthetic peptide, *ShK-186*, an analogue of the sea anemone *Stichodactyla helianthus* toxin (90, 91). Chabra et al. (91) have identified and characterized two other selected peptides, AcK1 and BmK1 from a large family of *ShK*-related peptides and showed that, they can preferentially inhibit K_v_1.3. *ShK-186* (Dalazatide) has recently passed phase 1 clinical trials and has shown to be well tolerated. A predicted range of drug exposure was achieved, and no serious adverse reactions were observed (https://clinicaltrials.gov/ct2/show/NCT02446340 **)**. In the phase 1b trial, 5-10 patients with plaque psoriasis, had improvements in target lesion score relative to baseline and 9 in 10 patients had improvements that were sustained for 4 weeks following the last dose (https://clinicaltrials.gov/ct2/show/NCT02435342). Phase 2 trials are planned to test efficacy in patients with SLE and efficacy trials in patients with multiple sclerosis and rheumatoid arthritis. In clinical studies modulation of cellular effector function by *ShK*-186 may constitute a novel treatment strategy for granulomatosis with polyangiitis with high specificity and less harmful side effects (92).

Li et al. (93) described a novel peptide ADWX-1 that is a selective K_v_1.3 blocker. ADWX-1 considerably reduced EAE in the rat model by selectively inhibiting CD4+CCR7− phenotype effector memory T cell activation. In addition, they also showed K_v_1.3 knockout mice were resistant to the development of multiple sclerosis-like syndrome. Peptide inhibitors of ion channels are not without their limitations. Due to their low molecular mass, peptides face rapid renal excretion. PEGylation and conjugated variants have been tested to increase circulation half-life hand the most effective and efficient method for retaining blood concentrations above the half-maximal inhibitory concentration (IC_50_) has been through subcutaneous injections, remaining at therapeutic serum concentrations for 2-7 days in rat and monkey models.

With K_v_1.3 being preferentially expressed in TEM cells consideration is given to the conjoint use of K_v_1.3 channel blockers with the Bacillus Calmette-Guerin (BCG) Tuberculosis vaccine to improve its efficacy (94). The vaccine on its own has a low and varied reported efficacy, despite a robust Th1 T cell response, thought to be due to the poor generation of cellular memory. However, if the TEM response is restrained, a greater TCM response would occur generating a larger pool of TCM cells from which TEM cells could later be produced, resulting in a longer-lasting and more robust immune memory, consequently improving the efficacy of the vaccine.

To date, the highest affinity K_v_1.3 inhibitors with the best K_v_1.3 selectivity are the sea anemone analogues engineered from the *ShK* peptide (e.g., *ShK*-186), the engineered scorpion toxin HsTx1[R14A] and the natural scorpion toxin Vm24 (95). These peptides inhibit K_v_1.3 in picomolar concentrations and are several thousand-fold selective for K_v_1.3 over other biologically critical ion channels (96). The increasing number of autoimmune diseases and the application of these potent inhibitors of K_v_1.3 hints at a rich source of new pharmacological tools and therapeutic leads.

### Anti-tumor response

Tumour-infiltrating lymphocytes are dysfunctional in situ, and yet, capable of stem cell–like behavior when taken out of that milieu. It is hypothesized that high levels of extracellular K^+^ in the tumour microenvironment triggers suppression of T cell effector function. With the reduced activity of K_v_1.3 and KCa3.1 exhibited in tumor-infiltrating lymphocytes of cancer patients due to factors such as adenosine and hypoxia, the ability of the T cells to infiltrate and have cytotoxic action on the tumor is consequently impaired, leading to a failure of immune surveillance. It has been suggested that K^+^ channel activators should be developed as a potential therapy for solid cancers. Understanding the mechanism of T cell dysfunction, and the role of cancer tissue-dependent factors in the regulation of ion channel function, may lead to novel approaches for cancer immunotherapy (22).

Zhu et al. (97) showed that alpha2-adrenergic receptor agonists can substantially improve the clinical efficacy of cancer immunotherapy by reducing cAMP levels and protein kinase A activity. Increased expression of Hvcn1 in tumours reduces cytosol acidification and facilitates the production of reactive oxygen species. The increased expression of this channel in cancers that are characterized by hypoxia and angiogenesis has led to proposing Hvcn1 antagonists as potential therapeutics (38).

### Inflammation

The TRPV1 receptor, also known as capsaicin or vanilloid receptor, is a transmembrane ion channel present on sensory neurons and is involved with the sensation of pain and “itch” (98). Capsazepine was first described as a competitive TRPV1 antagonist in 1990 and shown to reduce pain with a promising future for pain relief. Recent studies have shown a role in CD4+ T cell function and having an immunomodulatory effect. Numerous TRPV1 antagonists have been developed by pharmaceutical companies (87). In models of T cell–mediated colitis, TRPV1 promoted pathogenic T cell responses and intestinal inflammation. This raises the possibility that inhibition of TRPV1 could represent a new therapeutic strategy for restricting proinflammatory T cell responses (21).

There is increasing interest in the active crosstalk that occurs between nociceptor neurons and the immune system *via* ion channels to regulate pain, host defense, and inflammatory diseases. The discovery of the concept that reflex neuronal circuits control immunity provides a new mechanistic understanding and approach to infection, and inflammation. Pinho-Ribeiro et al. (99, 100) reported that lethal tissue destruction seen in streptococcal necrotizing fasciitis is the result of sensory neurons reflexively secreting calcitonin gene-related peptide (CGRP) that inhibits neutrophil migration. Treatment with Botulinum neurotoxin A (BoNT/A) and CGRP antagonist block neural suppression of CRGP release and allows the immune system to overcome the infection. Thus, there is an interplay between immune cells and neurons acting *via* ion channels. Immune cells/inflammation releases factors that modulate pain and thermal sensitivity. In turn, sensory neurons release neuropeptides and neurotransmitters from nerve terminals that regulate vascular, innate, and adaptive immune cell responses.

Recently, the Orai1-STIM complex was investigated as a target in acute and progressive Th17-mediated inflammatory kidney injury (101). Mouse models deficient in Orai1 or STIM1, when subjected to MHC-mismatched skin allografts, were unable to efficiently reject the grafts.

When the T cells from these mice were transferred to fully allogeneic mice, they had slower and attenuated acute graft-versus-host disease. This suggests that an Orai-STIM complex blocker may be more suited to use as an immunosuppressant.

### Other

The LRRC8C-STING-p53 signaling axis as a novel inhibitory pathway in T cells that controls T cell-mediated immune responses. The pharmacological suppression of LRRC8C may provide a novel approach to enhancing T cell function in the context of immunity to infection and antitumor immunity. LRRC8C is selectively enriched in T cells and its deletion is not lethal in mice, by contrast to the obligatory LRRC8A channel. Targeting LRRC8C may represent a more specific and safer approach to modulating T cell function (4). P2X7 channel inhibitors may be novel therapeutic agents for hypertension. Clofazimine, a medication used to treat leprosy is a K_v_1.3 channel blocker. In diseases such as multiple sclerosis, rheumatoid arthritis, and type 1 diabetes featured by pathogenic TEM cells which are characterized by K_v_1.3-high expression the use of this medication may be of value.

Therapeutic monoclonal antibodies (mAb) have revolutionised the management of many clinical diseases over the past few decades. Chimeric humanised and fully humanised mAb can now be made by recombinant engineering with tolerable short-term side-effects that are the mainstay of many auto-immune disorders. Unfortunately, mAb-based therapies targeting ion channels so far has not been developed for clinical use. Haustrate et al. (102) give a comprehensive review of mAb against ion channels, describe their mechanisms of action, and discuss their therapeutic potential.

## Conclusion

There is an abundance of ion channels expressed in lymphocytes that are important for development and function. With time the importance of these individual ion channels is becoming better recognized, and the interplay between each other, and the crosstalk that occurs is becoming better understood. New channels, and better understanding of function and mechanism of action, are being continuously recognised. It is amazing how different fluctuations in temporal expression and amounts of intracellular Ca^2+^ can play a pivotal role in directing crucial T cell functions through the activation and regulation of transcription factors “sensitive” to these changes. Based on the strength of stimulation and level of ion channel expression the immune response can be varied. Similarly astonishing is; (a) the recent recognition that ion channels may have other functions than their primary function of ion pores; (b) ion channels can contribute to the development of different T cell subsets; (c) as a corollary, T cell subtypes express different ion channel phenotypes, which allow; (d) specific T_EM_ and exact ion channels to target pathogenic T cells without compromising general systemic immunity; (e) the potential of developing new therapeutic drugs.

This overview examined the role played by ion channels in the function of T cells, in health and disease, and outlined future avenues for future pharmacological therapy. The targets include the Orai1-STIM complex because of its central role in Ca^2+^ influx, signaling, and T cell function, P2X7 and P2X4 channels, and K_V_1.3, KCa3.1 in the immunological fight against cancer. Research into ion channel expression, function and modulations in T cells is a growing field in immunology and there remains much to discover about the possible connections between ion channels, disease, and the development of new therapies.

## Author contributions

NM, design and conceptualisation of manuscript. Involved with writing, editing, and manuscript submission. DA, involved with drafting and revising the manuscript critically for important intellectual content. JP, Acquisition of references, involved with editing and reviewing the manuscript.
